# Novel Graphene-Based Materials as a Tool for Improving Long-Term Storage of Cultural Heritage

**DOI:** 10.3390/ma16093528

**Published:** 2023-05-04

**Authors:** George Gorgolis, Steffen Ziemann, Maria Kotsidi, George Paterakis, Nikos Koutroumanis, Christos Tsakonas, Manfred Anders, Costas Galiotis

**Affiliations:** 1 Institute of Chemical Engineering Sciences, Foundation of Research and Technology-Hellas (FORTH/ICE-HT), Stadiou Street, Platani, 26504 Patras, Greece; ggorgolis@iceht.forth.gr (G.G.); kotsidimaria@gmail.com (M.K.); gpaterakis@iceht.forth.gr (G.P.); nickkoutrou@iceht.forth.gr (N.K.); c.tsakonas@iceht.forth.gr (C.T.); 2 Department of Chemical Engineering, University of Patras, 26504 Patras, Greece; 3 Zentrum für Bucherhaltung GmbH (ZFB), Bücherstraße 1, 04347 Leipzig, Germany; ziemann@zfb.com (S.Z.); anders@zfb.com (M.A.)

**Keywords:** cultural heritage, temperature/humidity regulators, graphene, poly-vinyl alcohol

## Abstract

The very serious problem of temperature and humidity regulation, especially for small and medium-sized museums, galleries, and private collections, can be mitigated by the introduction of novel materials that are easily applicable and of low cost. Within this study, archive boxes with innovative technology are proposed as “smart” boxes that can be used for storage and transportation, in combination with a nanocomposite material consisting of polyvinyl alcohol (PVA) and graphene oxide (GO). The synthesis and characterization of the PVA/GO structure with SEM, Raman, AFM, XRD, Optical Microscopy, and profilometry are fully discussed. It is shown that the composite material can be integrated into the archive box either as a stand-alone film or attached onto fitting carriers, for example, those made of corrugated board. By applying the PVA/GO membrane this way, even with strong daily temperature fluctuations of ΔT = ±24.1 °C, strong external humidity fluctuations can be reduced by −87% inside the box. Furthermore, these humidity regulators were examined as Volatile Organic Compounds (VOCs) adsorbers since gas pollutants like formic acid, formaldehyde, acetic acid, and acetaldehyde are known to exist in museums and induce damages in the displayed or stored items. High rates of VOC adsorption have been measured, with the highest ones corresponding to formic acid (521% weight increase) and formaldehyde (223% weight increase).

## 1. Introduction

Tackling the degradation of cultural heritage requires a global effort. This is why the development of new nanomaterials and methods for the preservation of artwork is of high significance. Nano-science can serve conservation excellently because, in contrast to conventional materials like polymers applied in conservation today, engineered nanomaterials do not affect the chemical and physical properties of the artworks and have a low environmental footprint. Here, the aim of the work is the combination of paper conservation with advanced materials science and nanotechnology in order to provide solutions to temperature and humidity exposure, which are known problems encountered in small and medium-sized museums and galleries that contribute to the degradation of works of art either on display or in storage. The proposed solution entails the usage of “smart” packaging boxes filled with a composite material consisting of graphene oxide and polyvinyl alcohol. The composite material can be exploited either as a stand-alone film or as a coating applied to the corrugated board in the “smart” box. A systematic characterization of the as-obtained composite film by means of Scanning Electron Microscopy, Raman spectroscopy, Atomic Force Microscopy, Optical Microscopy and profilometry is presented here. The proposed material was tested inside a climatic chamber and found to be an efficient temperature and humidity regulator, and furthermore was found to have the ability to capture some of the most-known volatile organic compounds, like formic acid and formaldehyde, detected inside such (micro)-environments (museums, galleries, libraries, et al.).

## 2. Materials for Cultural Heritage Conservation

Important paintings, historic library collections, zoological preparations, certificates written on parchment, paper documents, or contemporary art—cultural assets are as different in their natures and meanings as they are in their chemical structures. Since the vast majority of such objects are not on display in controlled environments but in storage, a strong focus is placed on the influence of the storage climate on the materials’ chemistry in order to slow down the aging processes. As a result, maintaining adequate climatic conditions in warehouses or storerooms in order to conserve such movable cultural heritage (CH) objects is an important issue. The degradation of CH artifacts can seriously increase due to exposure to unstable temperatures, relative humidity (RH), light, and environmental pollutants [1]. As an example, acidic historic papers and documents containing iron gall ink are vulnerable to deterioration when exposed to temperature and humidity fluctuations [2,3]. Variations of ±10 %RH and ±5 °C in temperature at RH values below 65% are considered acceptable for paper-based objects in general [4]. However, this often cannot be applied in normal practice: valuable objects owned by museums and archives often have to be stored in climatically unsuitable locations (basements, boxes, drawers etc.).

One remedial action would be to install air conditioning, which is most reasonable but usually not affordable for small and medium-sized institutions. A plausible alternative to that is to create favorable conditions in each storage package through integration of active materials or modules. However, in addition to their obvious functions, these climate regulators must also meet some important requirements, some of which are that they should be non-toxic, thin/demand little space, easy to implement, easy to use, and cheap.

In general, temperature control might be challenging because it requires thick insulation panels like those already applied in the building sector. The implementation of such panels into enclosures would lead to an occupation of too much space inside the packages, thus expanding their dimensions and multiplying the required storage space. However, fluctuations in RH, which are also caused by changes in temperature, are easier to control, which demands fewer active materials. Known compositions for that purpose include CaCl_2_, silica gel, or diatomaceous earth, which lead to the drying of the surrounding atmosphere until the desiccant’s saturation [5]. However, those systems require some space and could damage CH objects that need higher values of RH because CaCl_2_ and SiO_2_ are primarily drying and will not return moisture to the environment when needed. Further, alkaline earth metal chlorides are known to release highly problematic HCl [6].

Graphene oxide (GO), a graphene derivative, is an abundantly available, low-cost carbon compound which is composed of a cluster of reactive oxygen functional groups [7]. GO can be synthesized from graphite or graphite oxide [8] by employing the Brodie, Staudenmaier, or Hummer’s methods or their modifications [9]. It can interact with a broad range of organic and inorganic materials [10], is highly hydrophilic, and can form stable aqueous dispersions to promote the assembly of macroscopic structures via simple and cheap solution processes. GO has been also exploited in adsorbing/sensing water vapor [11] thanks to the presence of abundant organic functional groups containing oxygen moieties [12]. Furthermore, GO has been reported to have the ability to capture volatile organic compound (VOC) molecules [13,14,15] because its rich oxygen-containing functional groups such as carbonyl, carboxyl, hydroxyl, and epoxy are catalytic in improving the vapor adsorption capability, thus enhancing the sensitivity [13]. The functional groups are critical in vapor adsorption kinetics by means of altering the electrical or optical properties of GO upon interaction with gaseous water [14], alcohols [15], and NH_3_ [16]. On the other hand, polyvinyl alcohol (PVA) is also considered as a hydrophilic material with high mechanical strength, low fouling potential, pH stability, and biocompatible characteristics [17]. PVA also offers a homogeneous matrix to each examined filler with strong interfacial interactions between them [18].

There are several reports in the literature on composite materials combining the properties of GO and PVA, especially for monitoring relative humidity and environmental pollutants. It has been reported that graphene flakes can change their crystal structure and be rotated when are efficiently impregnated in polymers [19], and, also, that the existence of edges in contact with the polymeric matrix enables dissociative chemisorption of oxygen [20]. Huang et al. have successfully prepared nanocomposite films of GO uniformly dispersed in a PVA matrix with significant improvements to the barrier properties for both oxygen and water vapor [21,22]. The full exfoliation, uniform dispersion, and high alignment in the PVA matrix and the strong interfacial adhesion between the GO nanosheets and the PVA matrix resulted in low rates of oxygen and water permeability with direct applications to the packaging industry. The reduction of oxygen permeability can be mainly ascribed to the reduced oxygen solubility in the PVA/GO composite film, as Kim et al. have described [23]. Apart from barrier properties like moisture and oxygen resistance, the thermal and mechanical properties are known to be improved as well as a consequence of the rigid structure and high aspect ratio of the exfoliated GO but also due to the strong interaction between PVA and GO [24]. However, a composite GO/PVA film has not ever been proposed as a regulator for humidity and pollutants existing in micro-environments.

This paper presents a solution to the aforementioned problem via implementation of PVA and GO modules into archive enclosures as a promising application.

## 3. Experimental Procedure

### 3.1. Synthesis of Graphene Oxide

Graphene Oxide (GO) was synthesized from natural graphite flakes (NGS Naturgraphit GmbH, Germany) via a two-step oxidation process, based on a modified Hummer’s method [11,25]. This involves a pre-oxidation step, followed by the final oxidation step where GO flakes were collected in an aqueous dispersion. In the first step, a flask was charged with 75 mL of concentrated sulfuric acid (H_2_SO_4_, 96%) and 10 g of natural graphite flakes were added. The reaction mixture was heated to 80 °C when 5 g of potassium persulfate (K_2_S_2_O_8_) and 5 g of phosphorus pentoxide (P_2_O_5_) were added. After stirring for 1 h at this temperature, the reaction mixture was left for 5 h at room temperature. At the end of this process, a dark blue solution was obtained. The reaction was terminated by carefully adding deionized water (DW), followed by vacuum filtration and washing with DW until the pH reached the pH of the DW. The pre-oxidized graphite was dried overnight under ambient conditions. This material was then subjected to oxidation via the modified Hummers method where the powder was added to 220 mL of H_2_SO_4_ (96%) under continuous stirring. Slowly, 26.7 g of potassium permanganate (KMnO_4_) was added to the mixture, keeping the reaction temperature below 20 °C. Thereafter, the mixture was heated at 40°C for 2 h, followed by the careful addition of 450 mL of DW. After 15 min, the reaction was terminated by adding 1.35 L of DW and 22 mL of hydrogen peroxide (H_2_O_2_, 30%). The bright yellow mixture was filtered and washed with a 10% *w/w* HCl solution in water to remove most of the metal ions. The solid product of this process was redispersed in DW and subjected to dialysis until the pH became the same as the pH of the DW. Finally, a mixture of single and few layers of GO was collected [11,25] after a combination of ultra-sonication at 75 W and centrifugation in the range of 3000 to 5000 rpm. Spectroscopic and morphological characterization techniques such as Raman, SEM, AFM, ATR, and XPS were employed in order to assess the quality of the produced material. The Raman spectrum of GO is reported in detail in the Supporting Information Section. Both SEM and AFM images indicated that the GO was well exfoliated (Figure S1B–D) with a maximum lateral size of 150 μm. Moreover, from the AFM measurements, the thickness of a single layer of GO was estimated at ~1.2 nm. The increase of the thickness of the GO, compared to a single layer of graphene (0.335 nm), is related to the presence of functional groups on the GO lattice [21,24,25]. On the other hand, more structural defects such as holes, cracks, and wrinkles are visible from AFM images. These defects are solely the result of the GO synthesis process, while the GO flakes in general are decorated by hydroxyl, carboxyl, and carbonyl functional groups [26]. Furthermore, from the ATR spectrum of Figure S2A, it can be deduced that the peak at ~1730 cm^−1^ corresponds to the vibrations along the C=O bonds of the epoxy groups, while the peak at ~1625 cm^−1^ corresponds to the asymmetric vibrations of the C=C bonds of the graphene lattice. The bending of the O–H bonds of the hydroxyl groups of the oxidized lattice appears at ~1380 cm^−1^ and the vibrations along the C–O bonds appear at ~1080 cm^−1^ [27]. The XPS analysis of the C 1s peak spectra of GO (Figure S2B) shows evidence for the presence of hydroxyls (-OH) and epoxides (C–O–C), which usually appeared in the main structure of the matrix, as well as carboxyl groups (-COOH), which were detected at the edges [28]. The relative atomic concentration of O/C was calculated as 0.6 by using the area ratio of the peak O 1s and C 1s.

### 3.2. Preparation of GO/PVA Films

**Figure 1:**
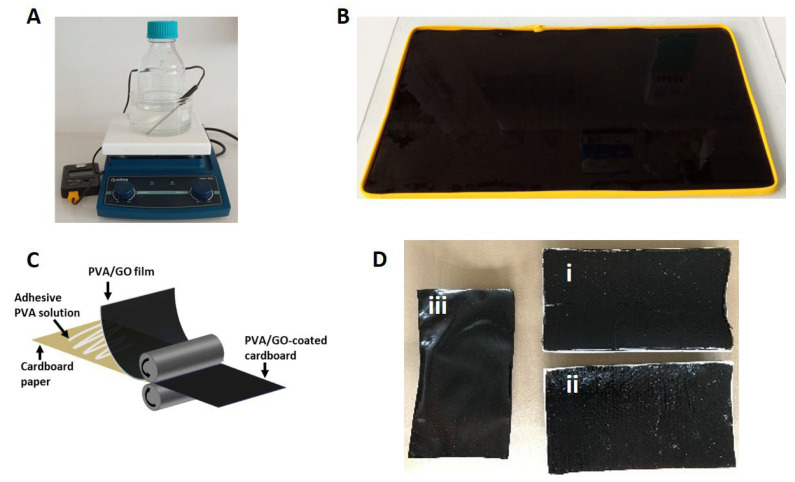
(**A**) Digital photo of the performed oil bath for PVA in water. (**B**) Drop-casting of the final solution onto the substrate. The size and shape of the substrate defines the dimensions of the material. (**C**) Schematic illustration of the roll-to-roll process [29] for transferring PVA/GO film onto cardboard. (**D**) PVA/GO-coated cardboards coated with the assistance of an adhesive PVA solution (i and ii) and a free-standing PVA/GO film (iii).

The novel graphene oxide/polyvinyl alcohol (GO/PVA) films, which were examined as relative humidity regulators, were synthesized from a mixture of aqueous solutions of PVA (10% *w*/*w* in deionized water) and GO (with a concentration equal to 20 mg/mL in deionized water). Initially, to dissolve the PVA in water, the solution was stirred in an oil bath at 90 °C for 4–5 h until a homogeneous polymeric matrix was formed (Figure 1A). After the solution was cooled down to room temperature, it was degassed under a vacuum for one hour. As for the GO solution, because of its high concentration, aggregations of GO flakes were observed. To address this issue, the GO solution was treated with a mortar and pestle to break down the aggregations and then was bath sonicated for several hours (4–5 h). Finally, the prepared solutions were mixed at a volume ratio of GO/PVA of 1:5. The mixture of PVA and GO is highly viscous, so intense stirring for one hour was required, followed by bath sonication for 2–3 h, to ensure homogeneous dispersion of the GO flakes inside the polymer. The final step of the process was the degassing of the prepared PVA/GO solution under a vacuum for one hour. The PVA/GO films were fabricated by drop-casting the final solution onto the desired substrates (Figure 1B). Two different configurations were prepared, a PVA/GO-coated cardboard and a free-standing PVA/GO film (Figure 1D). The free-standing PVA/GO film can be exploited either as a wrapping protective film for various objects or applied using the roll-to-roll method [29] as an additional protective coating on any substrate. To demonstrate the process of the roll-to-roll application of the PVA/GO film [29], a model cardboard substrate was coated with the hybrid film with the assistance of an adhesive PVA solution (Figure 1C).

### 3.3. Characterization Methods

SEM photos were taken using a LEO SUPRA 35 VP, while Raman spectra of the specimens were recorded using an InVia Reflex (Renishaw, UK) MicroRaman equipment using a 514 nm laser excitation. In all experiments, spectra were recorded at several points on each specimen using a Renishaw InVia Raman Spectrometer with a 1200 groove/mm^−1^ grating and a ×100 lens. The power of the laser beam was kept below 1 mW to avoid heating of the specimen. Raman spectra were baseline corrected and graphene peaks were fitted to Lorentzian functions. When graphene peaks were superimposed onto the peaks of the substrates, the necessary deconvolution process was applied. In this analysis, the Lorentzian components assigned to the substrates were held fixed, having had their parameters (position: full-width at half-maximum) evaluated from the spectra of the bare substrates.

Atomic Force Microscopy (AFM) images were collected in peak-force tapping mode (Bruker, Dimension-Icon), by using ScanAsyst-Air probes (silicon tips on silicon nitride cantilever, Bruker) with a 0.4 N m^−1^ nominal spring constant of the cantilever. X-ray diffraction measurements were performed with the assistance of a Bruker D8 Advance model diffractometer, while for Optical Microscopy, the inspection of the specimen was performed by using a ΝΙΚOΝ Eclipse L150 optical microscope. The roughness of the different substrates used in this study was measured with a profilometer (Bruker DektakXT 2D). The duration of the scan varies from 10 to 150 s and its length from 100 to 1000 μm while the force exerted by the stylus on the substrate is adjusted depending on how soft the sample is. Therefore, the lowest tip forces were applied on the examined substrates, which were fixed to the instrument base by using a minimum quantity of adhesive tape applied at the edge of the specimen. Each material was examined three times to derive an average roughness value. Surface roughness is expressed in terms of Rq (nm), that is the root mean square deviation of the roughness profile [30,31]:

(1) $$R_{q}=\sqrt{\frac{1}{l}\;\int_{0}^{l}Z^{2}\;\left(x\right)\mathrm{dx}}$$

where Ζ(x) is the height of the peak or depth of the valley and l is the length of the sample.

The XPS measurements were carried out in an ultra-high vacuum system (UHV), which consists of a fast entry specimen assembly, a sample preparation, and an analysis chamber equipped with a dual anode (Al/Mg) X-ray gun and an LH10 electron analyzer. The base pressure in both chambers was 1 × 10^−9^ mbar. An unmonochromatized MgK line at 1253.6 eV and an analyzer pass energy of 36 eV, giving a full width at half maximum (FWHM) of 0.9 eV for the Au 4f7/2 peak, were used in all XPS measurements. The XPS core level spectra were analyzed using a fitting routine, which can decompose each spectrum into individual mixed Gaussian–Lorentzian peaks after a Shirley background subtraction. The sample was mounted onto a Si substrate with dimensions of 1.5 × 1.5 cm^2^. Finally, the capacitance and impedance of the sensors were recorded by a computer-controlled LCR (E4980A, Agilent, CA, USA). Finally, an instrument for measuring the attenuated total reflectance (ATR, Golden Gate) was used for collecting the analogous spectrum for GO.

### 3.4. VOCs and Relative Humidity Adsorption Tests

The gas adsorption tests were conducted in static conditions in a closed glass desiccator with an excess of pollutant, using a saturated vapor stream at room temperature, as described elsewhere [13,14]. A total of 100 mL of each VOC, formaldehyde (37 wt.% in H_2_O), acetic acid (99 wt.% in H_2_O), formic acid (85 wt.% in H_2_O), or acetaldehyde (99.5 wt.% in H_2_O), was used as the pollutant source each time. For the humidity adsorption tests and creating particular relative humidity conditions, a saturated aqueous solution of specific salt (sodium chloride: 75%RH) and distilled water (100%RH) were used and placed in sealed containers at a stable temperature [11]. All samples were initially dried at 200 °C for two hours to remove the adsorbed humidity and weighed into a high accuracy balance to measure their dried mass. Afterwards, the films were loaded in a glass petri dish which was mounted inside the desiccator. The desiccator with the films and the fuming gas was stored inside a fume hood. Periodically, the mass of each sample was recorded by the weight meter, which was exactly next to the fume hood, minimizing the exposure of the specimens to the environment. The adsorption capacity of the prepared composite films was determined by calculating the percentage of weight change:

(2) $$A\%=\frac{\mathrm{last}\;\mathrm{weight}\;\mathrm{measurement}-\mathrm{initial}\;\mathrm{weight}\;\mathrm{measurement}}{\mathrm{initial}\;\mathrm{weight}\;\mathrm{measurement}}\;\cdot \;100$$

Once the composites reached the saturation point in the volatile gas adsorption, which corresponds to the maximum weight change, the weight of the specimens stabilized. In order to establish a baseline for the specimens’ saturation, after two consequent gravimetric measurements similar to the maximum observed value, the films were considered saturated.

Some of the most common commercial absorbent materials, activated carbon and silica gel, were used for a benchmark study. Activated carbon with an apparent density of 498 kg/m^3^ was purchased from Donau Carbon, while spherical silica gel with a particle size of 40–75 μm was purchased from Merck. These materials were dried and placed inside the desiccators with the same VOC sources. The masses of the absorbents were measured periodically until they reached saturation, and their maximum calculated adsorption capacities are presented in Section 4.2.

## 4. Results

**Figure 2:**
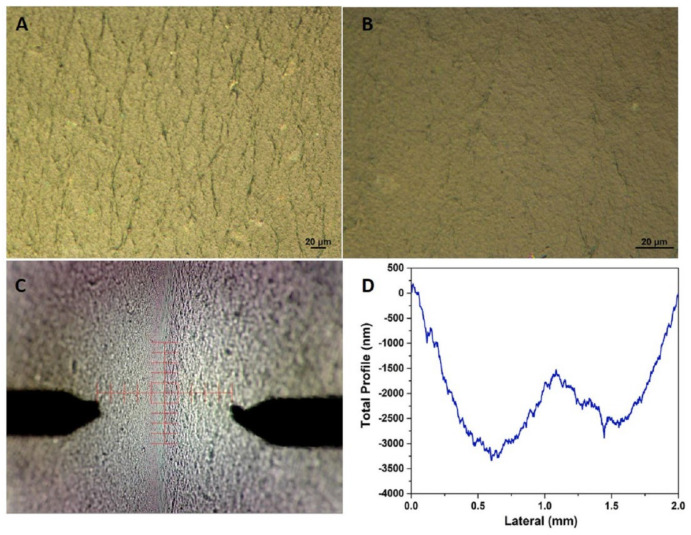
(**A**,**B**) Optical microscopy photos of the synthesized GO/PVA film at different scales, (**C**) Atomic Force Microscopy (AFM) image of the composite film, and (**D**) the measured surface profile of the specimen.

The specimens for the needs of the current study were prepared following the experimental route that has been already described. In Figure 1, the several consecutive steps and the finally resultant PVA/GO films, either as stand-alone film or deposited onto the target substrate, are presented. Optical images of the PVA/GO film shown in Figure 2A,B depict that the film has high homogeneity and flatness [32]. Figure 2C exhibits the tapping-mode AFM image of a PVA/GO film, confirming dense coverage of the GO nanosheets. It was demonstrated that the GO nanosheets are clearly well-dispersed in the PVA matrix. The thickness of the specimen was shown to increase when compared to the exfoliated 1.5-nm-thick single GO sheets (Figure S1), implying that the surface of the GO sheets was coated with PVA, and thus indicating that there were strong interfacial interactions between the two components [33]. Compared to the isolated individual GO platelets of Figure S1, the adjacent GO platelets are intralinked, creating a network due to the adsorption of the PVA molecules [34]. It is deduced that PVA functions as a bridge between the neighboring GO platelets along the basal plane direction. The 3D surface topography for the created PVA/GO film measured with profilometry (Figure 2D) displays a roughness (Rq) of 770 nm. The obtained value is higher than others reported for similar structures which lie in the range of 10–50 nm [35,36] and lower than others which reach up to ~100 μm [30].

**Figure 3:**
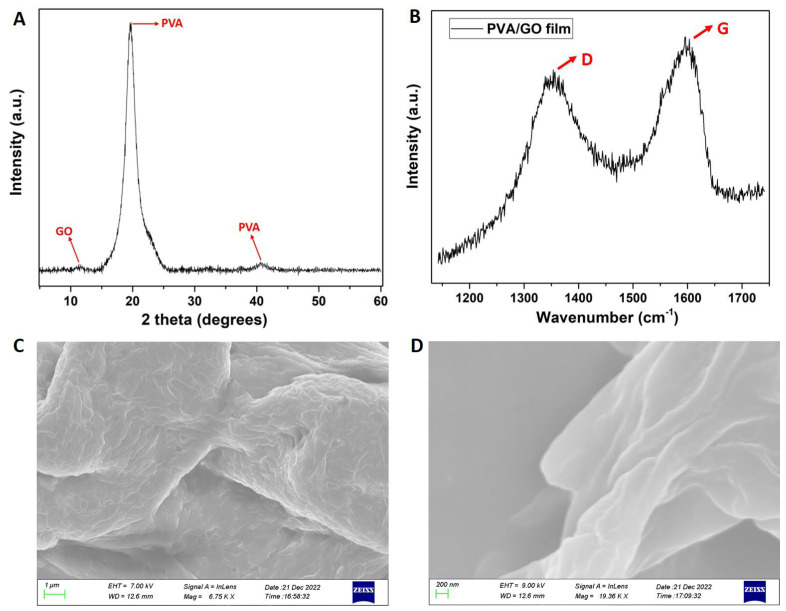
(**A**) XRD diagram, (**B**) Raman spectrum, and (**C**,**D**) SEM photos with different scales of the PVA/GO composite film.

From Figure 3A, which shows the XRD diagram, it can be observed that the characteristic diffraction peak of GO sheets appears at 2θ = 11.5° [31] and those of PVA are detected at around 2θ = 19.5° and 40.8° [19]. From the XRD pattern showing a not-intense GO peak, it can be deduced that the GO sheets were well-exfoliated and homogeneously dispersed in the PVA matrix while the crystallinity of PVA was slightly affected by the addition of GO component. From Figure 3B, the Raman spectrum of the PVA/GO film clearly reveals the presence of the D and G peaks attributed to GO [37,38]. In the GO spectrum (see also SI file), two peaks at ~1360 and ~1580 cm^−1^ assigned to the D band and G band were observed. The D band indicates the disorder in the structure of GO while the G band is related to the ordered sp^2^-bonded carbon [34]. It can be observed that PVA/GO composites show a similar spectrum as the neat GO, but with a slight shifting of the D and G bands to ~1355 and 1598 cm^−1^, respectively. The neat PVA has no characteristic Raman peak for the range of 1000–2000 cm^−1^, as was already reported [37,38]. Finally, the SEM photos depicted in Figure 3C,D show the microstructure of the PVA/GO composite films with no evidence of multi-layer stacks, a uniform GO dispersion into the PVA matrix [33], and a rather lamella aggregate [34].

### 4.1. Integration into Archive Boxes and Functionality

In this section, the above-described PVA/GO films—either as stand-alone films (“**PVA/GO plate**”) or deposited onto a carrier (“**PVA/GO board**”)—were examined with regard to their suitability for integration in archive boxes. Afterwards, their mode of humidity regulation was studied in an artificial environment that represents very drastic but realistic conditions at the same time.

**Figure 4:**
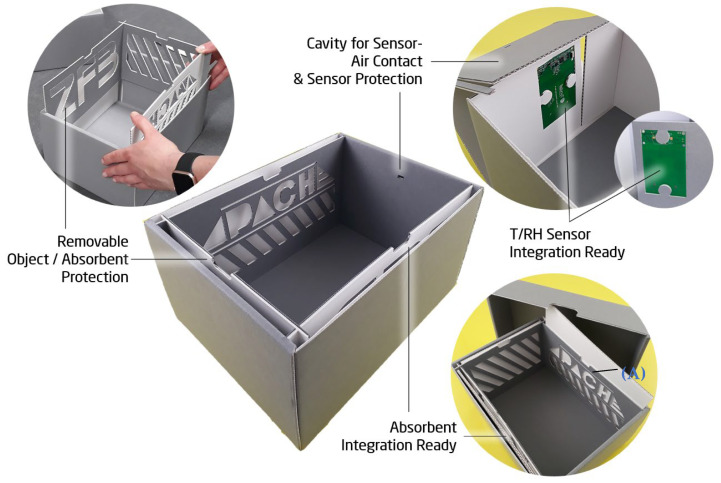
Prototype P2 of a novel archive box showing an exemplary method of absorbent (A) integration. The useable interior volume is V_Box_ = 24.5 cm × 17 cm × 14 cm (length × width × height).

**Figure 5:**
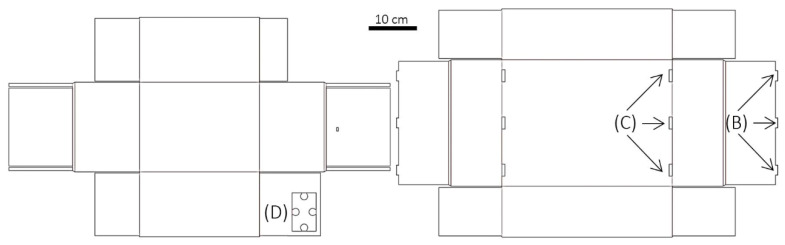
Comparison of CAD drawings of the novel ***P2*** box bottom (**left**) with a conventional constructed box bottom (**right**).

These PVA/GO modules (film or film+carrier, henceforth referred to as “regulators” or “modules”) can be individually plate-shaped and attached to the inside walls of the box. When applied in this way, a protection between the absorbent and object must be installed to prevent direct contact. For that reason, a protective cover is introduced with enough vent openings for air circulation. The so-created compartments between the protective cover and box wall give tight and stable supports for a plate-shaped regulator module. Figure 4 shows the enclosure prototype ***P2***, representing an optimized structure regarding airtightness and stability when compared to conventional archive box constructions (see Figure 5).

The lid has only a slightly larger basal plane than the base, resulting in a very tight closure. In the bottom and lid, planar inlays made of thicker corrugated board prevent the inwardly folded flaps from opening, which gives stability without using notches for fastening. This shall prevent an additional convective flow of volatiles into the box to avoid the intrusion of pollutants and a too-early absorbent deactivation. As Figure 5 illustrates, most of the conventional folded box constructions exhibit locking elements (B) that have to snap into notches (C) to create a stable joint that finally assembles the folded bottom/lid. Convection through these openings would reduce the possibly long lifespan of the regulating elements and would lead to a faster saturation of inflowing pollutants. In P2, one short side can be left blank, without compartments for absorbers. This is due to the intended integration of NFC sensor transponders to create a “smart” archive box, also shown in Figure 4 [39]. A cavity that gives the best possible sensor–air contact will need access to the inner microclimate and should not be covered by regulators. In the presented optimized construction, the sensor transponder is fixed with four mounting supports derived from a puzzle-like cutout (Figure 5D). The fastening flap that exhibits the sensor cavity simultaneously acts as a cover, which protects the sensible electronic components from direct contact with the object.

**Figure 6:**
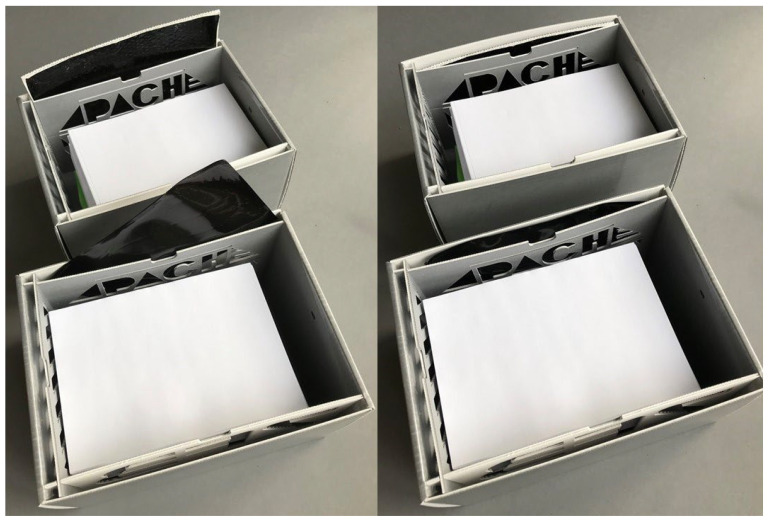
P2C boxes filled with paper and equipped with a PVA/GO board (**top**) and with a PVA/GO plate (**bottom**).

Figure 6 shows how the PVA/GO regulators are inserted into box P2C for experimental evaluations. From a design point of view, P2C is the same enclosure as P2, but is composed of coated corrugated board (the coating consists of a PET/EVAC [polyethyleneterephthalate/ethylene vinyl acetate] copolymer). To represent the realistic situation of an absorbent encased in a filled archive box, the modules have been put into coated P2C boxes filled with paper. The filling of each box with paper material is simulated by addition of DIN A5 paper sheets having a mass of m_Obj_ = 2450 g and an overall volume of V_Obj_ = 21 cm × 14.9 cm × 10.4 cm = 3254.2 cm^3^. Afterwards, the boxes were closed and subjected to artificial cyclical (daily) T and RH changes in a climate chamber. The exterior (climate chamber) and interior (inside the boxes) values for T and RH were monitored. It should be noted that the applied values for temperature and relative humidity are in a range that represents very harsh conditions which are not usually found in museums and archives. However, these external conditions led to the most evaluable results, as the differences in interior relative humidity between the boxes became clearer this way.

**Figure 7:**
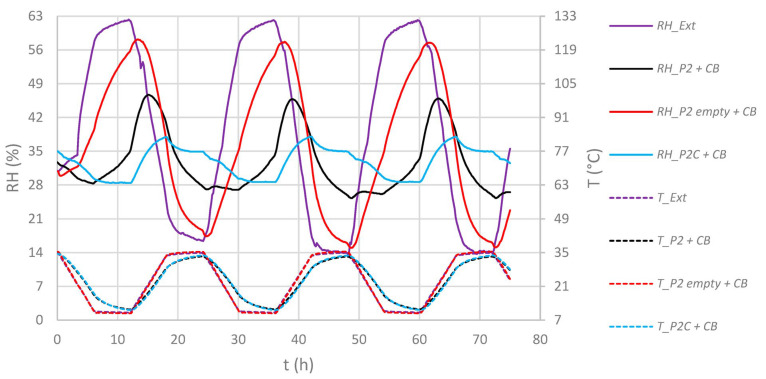
Time dependent T/RH diagrams outside (“Ext.”) and inside paper-filled (except “empty”) P2 or P2C enclosures. Solid lines: RH, dotted lines: T.

At first, an empty and paper-filled P2 box and a paper-filled P2C box were stored inside the climate chamber, leading to a T/RH profile as presented in Figure 7. As a reference, sheets of corrugated board of the same size compared to the regulator modules were inserted, which is referred to as “CB.” This represents the use of no regulator but with the same volume demand.

**Figure 8:**
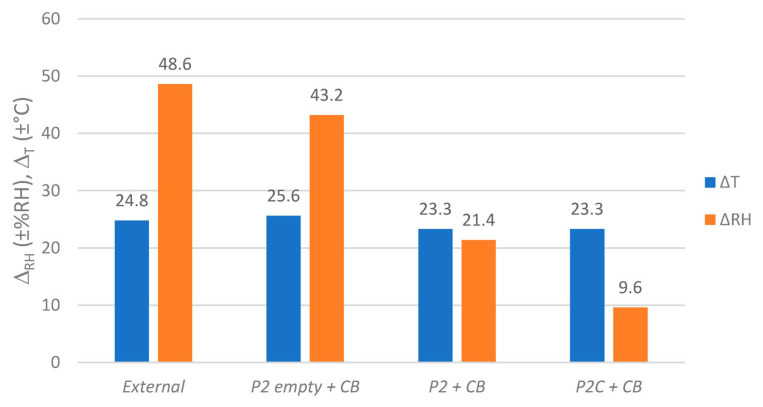
Humidity and temperature spans (Δ_RH_ and Δ_T_) outside and inside paper-filled (except “empty”) ***P2*** or ***P2C*** enclosures. Values based on fluctuations as shown in Figure 7.

As it becomes clear at the differences in RH span values Δ_RH_ inside the boxes from Figure 8, a strong attenuation effect in these conditions can be attributed to the contained stack of paper and the coating of the corrugated board itself. The paper stack as such also contributed to a small decline in Δ_T_, which is evidence for an absorption of thermal energy by the paper. This reduced Δ_T_ of 23.3 °C is causal for the remaining ±9.6 %RH inside ***P2C***, since the coating on the corrugated board shields the box’s interior volume from inflowing RH by blocking the diffusion-open surfaces. This is the minimum condition that must be met for regulators to function inside archive boxes, as the following experiment describes.

**Figure 9:**
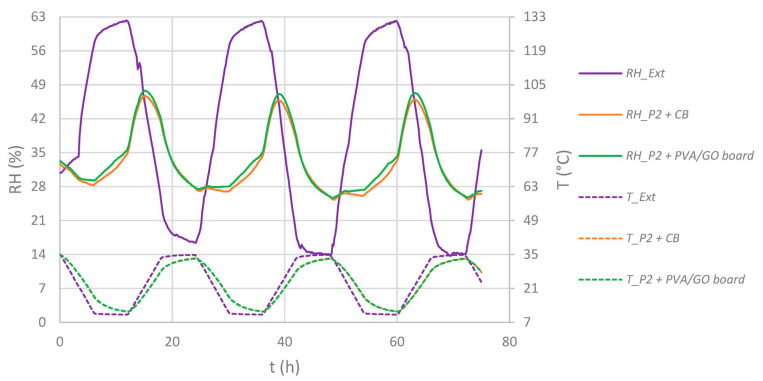
Time-dependent T/RH diagrams outside (“Ext.”) and inside paper-filled ***P2*** enclosures equipped with regulators (except “CB”). Solid lines: RH, dotted lines: T.

**Figure 10:**
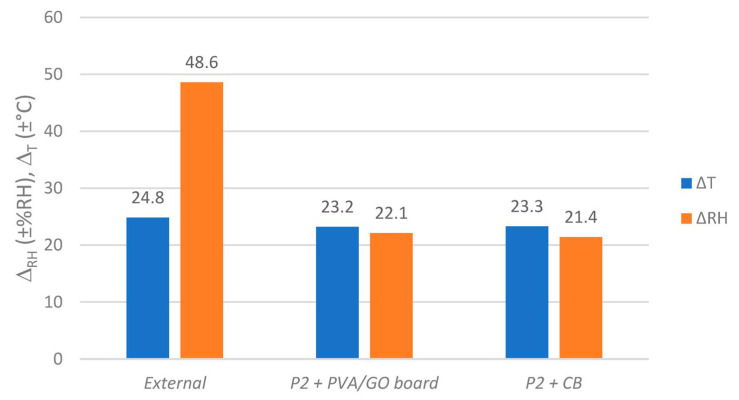
Humidity and temperature spans (Δ_RH_ and Δ_T_) outside and inside paper-filled ***P2*** enclosures equipped with regulators (except “CB”). Values based on fluctuations as shown in Figure 9.

When humidity regulators were added to the paper-filled, uncoated ***P2*** boxes, little effect could be seen on the RH scale when compared to the case “without regulator” (*P2 + CB*), see Figure 9 and Figure 10. The relative humidity inside the boxes fluctuated with spans of ±21.4 to ±22.2 %RH, being influenced by external RH and T fluctuations.

**Figure 11:**
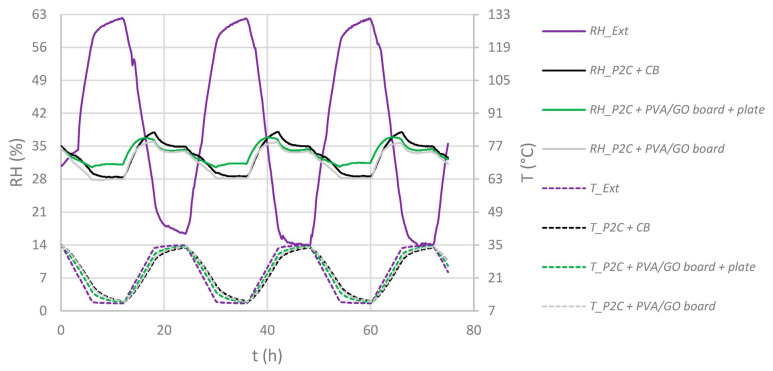
Time dependent T/RH diagrams outside (“Ext”) and inside paper-filled ***P2C*** enclosures equipped with regulators (except “CB”). Solid lines: RH, dotted lines: T.

**Figure 12:**
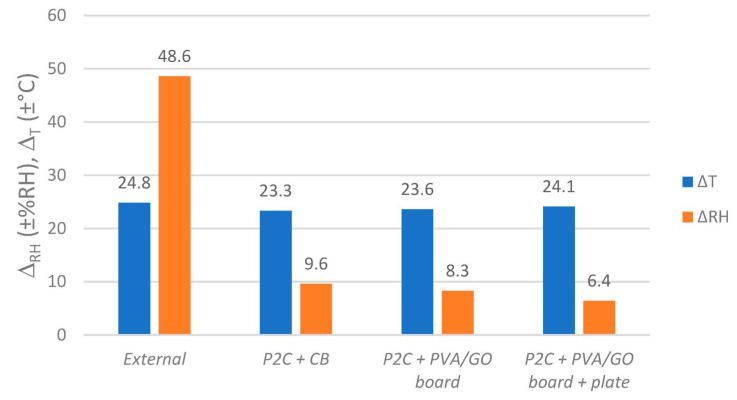
Humidity and temperature spans (Δ_RH_ and Δ_T_) outside and inside paper-filled ***P2C*** enclosures equipped with regulators (except “CB”). Values based on fluctuations as shown in Figure 11.

As a result, the regulator module PVA/GO board in this setup could not attenuate the inflowing humidity fluctuations and those resulting from the warming and cooling stack of paper at the same time. To evaluate the regulators’ potential for attenuating humidity fluctuations resulting from water-containing objects, the same experimental assessment was carried out in ***P2C*** boxes (see Figure 11 and Figure 12).

In general, the insertion of regulator modules led to a further decrease of RH fluctuation Δ_RH_ inside the paper-filled boxes when compared to the reference “*P2C + CB*” (±9.6 %RH). By applying one PVA/GO board, this value decreased to ±8.3 %RH. However, the current strongest additional fluctuation reduction was measured by applying the PVA/GO membrane as a coating on corrugated board and as a pure plate at the same time (Δ_RH_ = ±6.4 %RH). This means that in this paper-filled active archive box, even with strong daily temperature fluctuations of Δ_T_ = ±24.1 °C, strong external humidity fluctuations can be reduced by −87%. By applying the regulators *PVA/GO board + plate*, a further reduction of −33% in RH span was recorded when compared to the (inactive) reference CB without the used film swelling as a consequence of the humidity exposure. Additionally, neither film nor film-coated carriers showed any irregularities in structure uniformity or adhesion. This was determined after carrying out several experiments presented in the current study and after storage in a warehouse for one year. Additionally, the films seem quite resistant towards scratching, even though there are some signs of use. With a total 18.47 g of active material, a very small amount of material is necessary to achieve a noticeable effect under very harsh environmental conditions.

### 4.2. Sorption Tests of the GO/PVA Film

The fabricated composite films were tested for sorption of formaldehyde (CH_2_O), acetic acid (CH_3_COOH), formic acid (CH_2_O_2_), and acetaldehyde (C_2_H_4_O), which are the most common pollutants in facilities with artworks, as well as water vapor (relative humidity). These gaseous pollutants are emitted by paints, adhesives, furniture, building materials, etc. and have been proved to be significantly harmful after extended and continuous exposure to both humans and artworks [40,41,42].

**Figure 13:**
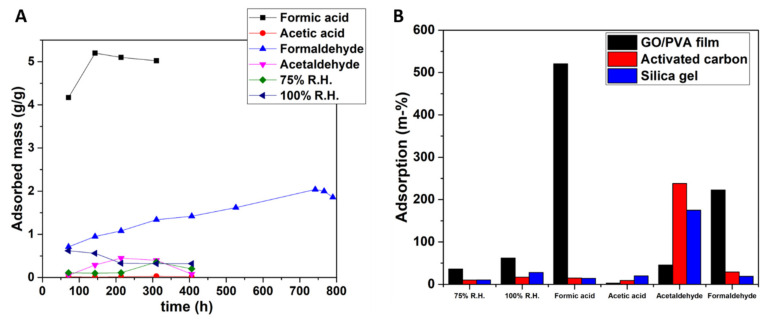
(**A**) Sorption isotherms (time-dependent adsorbed mass) of the GO/PVA film for the examined VOCs and relative humidities and (**B**) Rates of maximum adsorbed amount for the VOCs and different humidities in a static environment at room temperature for the examined GO/PVA film and commercial absorbers (activated carbon and silica gel).

It is important to note here that when developing absorbents for polar pollutants, in this work, all the examined gases and water are polar molecules, especially if selectivity for certain absorbates is needed. In effect, surface functionality becomes more important than pore volume or pore structure. GO interacts with polar VOCs while graphene prefers hydrophobic VOCs. Hence, the proper balance between hydrophobicity, surface area, and functionality should be considered in order to design an efficient adsorbent for VOCs. Except for the polarity of the examined VOCs, the presence of π bonds and hydrogen bonds in their structure, as well as their electron geometry, play a significant role in selective adsorption [14]. It is evident that different adsorption mechanisms may act simultaneously, which results in selective sorption of pollutants [13]. From the sorption isotherms of Figure 13A, it can be concluded that the GO/PVA film shows a remarkable adsorption of formic acid and formaldehyde. From Figure 13B, it is clearly evident that the as-obtained films have the ability to capture water molecules with adsorption rates equal to 36% and 62% for 75% R.H. and 100% R.H., respectively, denoting their hydrophilic character. Additionally, from this figure it is clear that the films exhibit selective adsorption of formic acid. The adsorption of formaldehyde and acetaldehyde follow, while the adsorption of acetic acid is the lowest. For the interpretation of the above observations, it seems that the interaction mechanisms between the pollutants and the films are mainly surface complexation, π-π stacking, and electrostatic interactions [14]. The nanosheets of GO are graphene-like sheets decorated with oxygen-containing functional groups. These functional groups play the role of active adsorption sites for polar molecules. By comparing the polarity of the examined VOCs, formic acid and acetic acid have the highest (both have a topological polar surface area equal to 37.3 Å^2^). Furthermore, both these molecules can form hydrogen bonds and π-π stacking with the conjugated π-network of GO. Therefore, the selectivity of formic acid over acetic acid by the examined films can be attributed to the electron geometry, since the formic acid has a planar configuration (trigonal planar) resulting in lower adsorption resistance compared to acetic acid (1-tetrahedral, 2-trigonal planar, 3-bent). It is known that the binding energy during the adsorption is primarily determined by the atom which is closer to the adsorbing site, so the smaller the distance between them, the higher the attraction. As for formaldehyde, it has the ability to form hydrogen bonds and contains a π bond, which contribute to its interaction with GO, yet because of its planar configuration, it exhibits lower polarity than the previously mentioned molecules. It is worth mentioning that the as-prepared GO/PVA film demonstrates higher adsorption for 75% and 100% RH formic acid and formaldehyde when compared to the commercial absorbers, while activated carbon and silica gel are superior for the cases of acetic acid and acetaldehyde. Regarding the comparison of the GO/PVA film to other pure GO or pure PLA films for VOC adsorption, it is denoted that for the first category, the synthesized material has comparative [13] or even better performance [14], even though both works are referred to as reduced GO structures which enhance VOC adsorption. For the latter category, a direct comparison cannot be made, since in the literature [17], pure PLA films are normally used for sensing or adsorption detected by specific sensors and not for VOC adsorption by gravimetric methods used herein.

It is also worth mentioning that the GO/PVA films can return to their initial dried state after reaching the maximum adsorbed material capacity. It has been observed that after placing the films inside a fume hood for 24 h with moderate ventilation, the weight of the samples reverts to the original value prior to exposure to VOCs and the relative humidity. This observation paves the way for the examination of the reusability of these materials in future.

These results show the multi-functional characteristics of these composite materials, paving the way for their immediate exploitation in cultural heritage protection.

## 5. Conclusions

In conclusion, a “smart” storage enclosure was presented and proved to function as a self-humidity-regulating archive box when equipped with graphene oxide (GO)/polyvinyl alcohol (PVA) composite films. The easy-to-apply and low-cost nanocomposite membrane makes the shown application very attractive for small and medium-sized museums, libraries, or archives. GO is well-dispersed in the PVA matrix and the resultant composite material can reduce the external humidity fluctuations by −87%, even with strong daily temperature fluctuations of ΔT = ±24.1 °C. Additionally, the composite was also found to be a good adsorber of VOCs with especially high rates for formic acid and formaldehyde, the latter being a major concern regarding CH degradation [43,44]. Apart from these two VOCs, acetaldehyde is adsorbed with a rate of a 45% weight increase, while relative humidities of 75% and 100% RH induced from saturated salt solutions are adsorbed with 36% and 62% weight increases, respectively. To conclude, this work has shown that the usage of graphene-related materials offers added protection against humidity and VOCs for artworks in storage in designated boxes. The application developed here is financially viable and ideal for small museums and art galleries.

## Data Availability

The data presented in this study are available on request from the corresponding author. The data are not publicly available due to usage for future publications.

## Supplementary Materials

The following supporting information can be downloaded at: https://www.mdpi.com/article/10.3390/ma16093528/s1, Figure S1: (A) Raman spectrum of produced GO, (B) SEM photo for GO sheets, (C) Atomic Force Microscopy (AFM) images of single layer GO flakes, and (D) height profile of the GO flakes with a thickness of 1.25 nm; Figure S2: Spectroscopic characterization of GO via (A) ATR spectrum [27] and (B) XPS [11].
