# Supplementary material for: Novel Graphene-Based Materials as a Tool for Improving Long-Term Storage of Cultural Heritage
Source: Materials (Basel). 2023 May 4;16(9):3528. doi: 10.3390/ma16093528 (PMC10180220; doi:10.3390/ma16093528)
Supplement: Supplementary file 1 [file materials-16-03528-s001.zip › materials-2340493-supplementary.pdf]

# Novel Graphene-Based Materials as a Tool for Improving Long-Term Storage of Cultural Heritage

**George Gorgolis [1,2], Steffen Ziemann [3], Maria Kotsidi [1], George Paterakis [1,2], Nikos Koutroumanis [1], Christos Tsakonas [1], Manfred Anders [3] and Costas Galiotis [1,2,*]**

[1] Institute of Chemical Engineering Sciences, Foundation of Research and Technology-Hellas (FORTH/ICE-HT), Stadiou Street, Platani, 26504 Patras, Greece; ggorgolis@iceht.forth.gr (G.G.); kotsidimaria@gmail.com (M.K.); gpaterakis@iceht.forth.gr (G.P.); nickkoutrou@iceht.forth.gr (N.K.); c.tsakonas@iceht.forth.gr (C.T.)
[2] Department of Chemical Engineering, University of Patras, 26504 Patras, Greece
[3] Department of Chemical Engineering, Zentrum für Bucherhaltung GmbH (ZFB), Bücherstraße 1, 04347 Leipzig, Germany; ziemann@zfb.com (S.Z.); anders@zfb.com (M.A.)
* Correspondence: c.galiotis@iceht.forth.gr or galiotis@chemeng.upatras.gr

The Raman spectrum of GO (Figure S1A) comprises several frequency bands, each one assigned to a certain structural configuration[25]. The main Raman peak is the so-called G peak, located at 1580 $cm^{-1}$ and is related to the $E_{2g}$ mode and the in-plane stretching of C=C bonds. The most prominent "disorder" Raman peak is the so-called D peak, appearing at about 1360 $cm^{-1}$, is related to the hexagonal ring breathing mode, the intensity of which is associated with the presence of either structural defects or the crystal edges of the lattice [28]. The 2D peak at 2680 $cm^{-1}$ is the second-order overtone of the D peak, which requires two-phonon scattering for activation, while the other two peaks at ~2950 $cm^{-1}$ and ~3100 $cm^{-1}$ correspond to the overtone of the D and G peaks and the G peak, respectively [29]. The two main Raman peaks of GO (G and D) are very sensitive to small changes in the $sp^3/sp^2$ ratio. Additionally, the intensity ratio of these two peaks, $I_D/I_G$, is correlated with the changes of the $sp^3/sp^2$ ratio and can be used for the characterization of GO. By analyzing a sufficient number of the Raman spectra, the intensity ratio $I_D/I_G$ for the GO was found to be 0.97 ± 0.01.

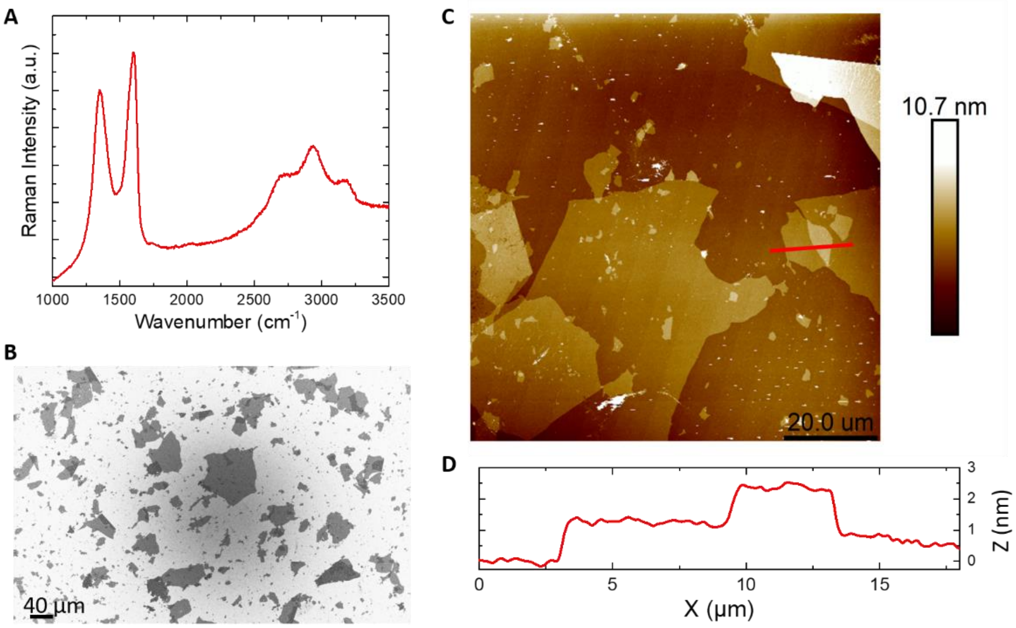


**Figure S1.** (**A**) Raman spectrum of produced GO, (**B**) SEM photo for GO sheets, (**C**) Atomic Force Microscopy (AFM) images of single-layer GO flakes, and (**D**) height profile of the GO flakes with a thickness of 1.25 nm.

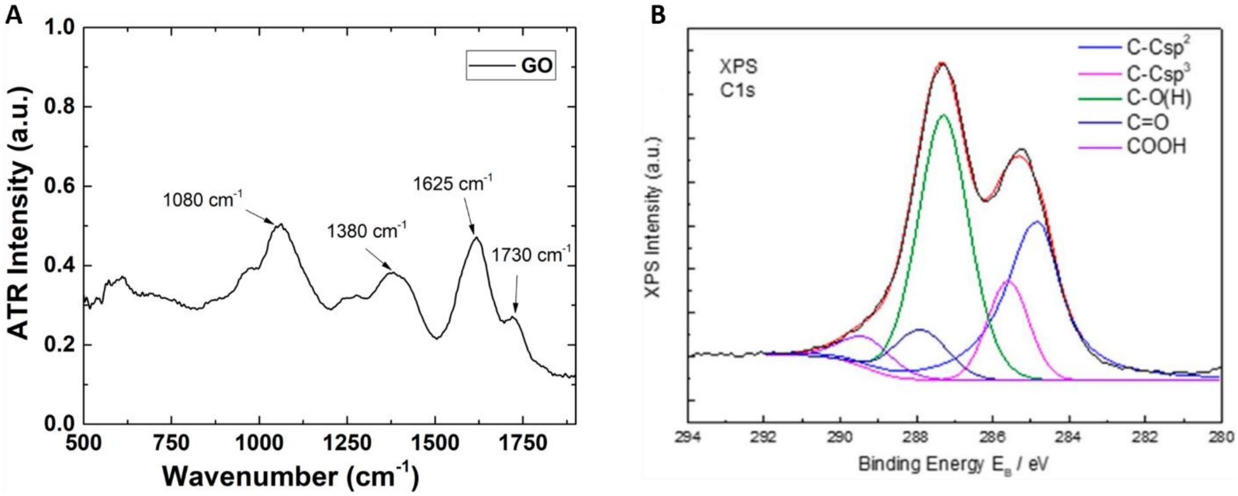


**Figure S2.** Spectroscopic characterization of GO by (**A**) ATR spectrum [4], and (**B**) XPS [5].

## References

25. Sygellou, L.; Paterakis, G.; Galiotis, C.; Tasis, D. Work Function Tuning of Reduced Graphene Oxide Thin Films. *J. Phys. Chem. C* **2016**, *120*, 281–290. https://doi.org/10.1021/acs.jpcc.5b09234.

28. Ferrari, A.C.; Meyer, J.C.; Scardaci, V.; Casiraghi, C.; Lazzeri, M.; Mauri, F.; Piscanec, S.; Jiang, D.; Novoselov, K.S.; Roth, S.; et al. Raman spectrum of graphene and graphene layers. *Phys. Rev. Lett.* **2006**, *97*, 187401. https://doi.org/10.1103/PhysRevLett.97.187401.

29. Das, A.; Chakraborty, B.; Sood, A.K. Raman spectroscopy of graphene on different substrates and influence of defects. *Bull. Mater. Sci.* **2008**, *31*, 579–584. https://doi.org/10.1007/s12034-008-0090-5.

20. Schröder, U.A.; Grånäs, E.; Gerber, T.; Arman, M.A.; Martínez-Galera, A.J.; Schulte, K.; Andersen, J.N.; Knudsen, J.; Michely, T. Etching of graphene on Ir(111) with molecular oxygen. *Carbon N. Y.* **2016**, *96*, 320–331. https://doi.org/10.1016/j.carbon.2015.09.063.

12. Paterakis, G.; Vaughan, E.; Gawade, D.R.; Murray, R.; Gorgolis, G.; Matsalis, S.; Anagnostopoulos, G.; Buckley, J.L.; O'Flynn, B.; Quinn, A.J.; et al. Highly Sensitive and Ultra-Responsive Humidity Sensors Based on Graphene Oxide Active Layers and High Surface Area Laser-Induced Graphene Electrodes. *Nanomaterials* **2022**, *12*, 2684.
